# Measuring Caregiver Person-Centered Communication for Persons With Dementia via Video Observations

**DOI:** 10.1093/geroni/igaa057.495

**Published:** 2020-12-16

**Authors:** Ying-Ling Jao, Carissa Coleman, Kristine Williams, Diane Berish, Wen Liu, Amy Berkley, Yo-Jen Liao

**Affiliations:** 1 Pennsylvania State University, University Park, Pennsylvania, United States; 2 University of Kansas, Kansas City, Kansas, United States; 3 University of Kansas, Lawrence, Kansas, United States; 4 University of Iowa, Iowa City, Iowa, United States

## Abstract

Communication is fundamental for daily care activities in nursing homes (NHs). Second-by-second behavioral coding of video observations is an ideal approach to examine the interactive nature of communication but requires a reliable coding scheme. Recent studies have adapted the Peron-Centered Behavioral Inventory (PCBI) and Task-Centered Behavioral Inventory (TCBI) to analyze caregiver communication during mealtime interactions, but their use for coding general daily caregiving activities has not been widely evaluated. This pilot study adapted the PCBI and TCBI of video observations and determined their inter-rater reliability for measuring caregiver verbal communication with persons with dementia (PwD). We analyzed videos from a randomized controlled trial of an intervention to improve caregiver communication in NHs. We selected one 1-minute segment from 12 videos that included interactions of caregiver-resident dyads. One research assistant transcribed caregivers’ verbal communication and segmented the communication into utterances. Two other research assistants independently coded each utterance using the adapted PCBI and TCBI. The coding scheme was expanded by modifying the existing operational definitions, adding three new codes, and developing a coding decision guide. Residents were Caucasian (100%), mean age 86 years with dementia and resistive behaviors. The adapted PCBI and TCBI had an inter-rater reliability of Kappa=0.656 (p<.001) across the 12 videos. Overall, our adapted PCBI and TCBI showed substantial inter-rater reliability. The results support the use of our adapted PCBI and TCBI to distinguish between person-centered and task-centered communication in video observations, which, in turn, allows for sequential analysis to examine the impact of caregiver communication on PwD.

